# Comparison of Auditory Stream Segregation Abilities and Cerebral Asymmetry in Processing Speech in Noise in Carnatic Musicians, Bharatanatyam Dancers, and Non-Trained Individuals

**DOI:** 10.3390/brainsci16020200

**Published:** 2026-02-07

**Authors:** Sreeraj Konadath, Aysha Nida, Praveen Prakash, Vijaya Kumar Narne, Sunil Kumar Ravi, Reesha Oovattil Hussain

**Affiliations:** 1 Department of Medical Rehabilitation Sciences, College of Applied Medical Sciences, King Khalid University, Abha 61481, Saudi Arabia; 2 Department of Audiology, All India Institute of Speech and Hearing, University of Mysuru, Mysuru 570006, India; 3 Department of Audiology, JSS Institute of Speech and Hearing, Karnatak University, Dharwad 580007, India

**Keywords:** auditory stream segregation, spectral profile analysis, speech-in-noise perception, cerebral asymmetry, carnatic musicians, Bharatanatyam dancers

## Abstract

**Highlights:**

**What are the main findings?**

- Long-term musical and dance training is associated with enhanced central auditory processing, indicating more efficient cortical integration of complex acoustic information.
- Trained individuals exhibit reduced hemispheric asymmetry and superior auditory performance, consistent with experience-driven auditory neuroplasticity.

**What are the implications of the main findings?**

- Musical and rhythmic movement–based training may promote adaptive neuroplastic changes in central auditory pathways that support auditory stream segregation and speech-in-noise perception.
- Training–related reductions in hemispheric asymmetry highlight the potential role of sensorimotor–auditory experiences in strengthening bilateral auditory–cognitive networks relevant to brain health.

**Abstract:**

Aim: This study compared spectral profile analysis thresholds, speech-in-noise perception, and cerebral asymmetry among Carnatic musicians, Bharatanatyam dancers, and non-trained individuals and examined the influence of training duration on these measures. Method: A total of 105 right-handed adults (18–30 years) with normal hearing were divided into Carnatic musicians (n = 35), Bharatanatyam dancers (n = 35), and non-trained controls (n = 35). Spectral stream segregation was measured using the spectral profile analysis task, and speech-in-noise perception was evaluated using the Kannada QuickSIN under right, left, and binaural conditions. Cerebral asymmetry was derived from the Laterality Index. As data were non-normally distributed, non-parametric tests were used. Results: Significant group differences emerged for spectral profile thresholds, with dancers outperforming musicians and controls. Both trained groups showed superior speech-in-noise performance compared to non-trained individuals across all listening conditions, though no differences were observed between musicians and dancers. Non-trained listeners displayed a clear right-ear advantage, whereas trained groups showed minimal or no hemispheric asymmetry. Training duration negatively correlated with selected spectral profile thresholds in both trained groups and with binaural SNR-50 in dancers, indicating training-related auditory enhancement. Conclusions: Musicians and dancers demonstrate better spectral discrimination, improved speech-in-noise perception, and reduced cerebral asymmetry compared to non-trained peers. These findings underscore training-induced auditory neuroplasticity and suggest that long-term engagement in music or dance promotes efficient auditory processing and greater bilateral hemispheric involvement.

## 1. Introduction

Emerging in infancy and continuing throughout life, the human auditory system develops the capacity to navigate complex acoustic environments by segregating and organising overlapping sounds into meaningful auditory streams, a process referred to as Auditory Scene Analysis (ASA) [1]. ASA enables the brain to distinguish between concurrent sounds, organising them into perceptual units called auditory streams [1]. It refers to a perceptual process by which similar sounds are grouped, and different ones are segregated by the central auditory nervous system [2]. Stream segregation, a critical aspect of ASA, refers to the process by which the brain separates overlapping or successive sounds into distinct auditory streams, using both simultaneous and sequential grouping of auditory signals [1]. This process is essential in everyday listening, where multiple sound sources are often present at once. In these complex auditory environments, individuals must separate and organise sounds into distinct auditory streams, enabling them to focus on specific sounds amid the surrounding noise [3].

Both spectral and temporal cues are essential for analysing auditory scenes. Spectral cues, such as spectral separation and profiling, and temporal cues, including separation, ordering, and regularity, contribute to this process [4,5,6]. Among these, spectral profile analysis is one of the primary cues that help in auditory stream segregation. This ability is crucial, as it enables the perception of changes in intensity patterns across different frequencies, aiding in the segregation of auditory streams [7]. To achieve this, the brain employs certain heuristic processes that rely on identifying regularities and patterns in incoming sounds from different sources. By analysing these cues, the brain is able to separate and organise auditory information, forming distinct mental representations for each sound source, thus influencing our ability to perceive pitch, tone quality, volume, and spatial location [1,2,8].

Extensive research shows that musical training induces plastic changes in both subcortical and cortical auditory systems, leading to enhanced auditory processing [9,10]. Over time, these neural adaptations translate into measurable behavioural advantages, with musicians often excelling in tasks that demand rapid auditory discrimination, fine temporal resolution, and effective speech perception in noise [11,12,13,14]. They also exhibit heightened sensitivity to subtle variations in pitch, timing, and loudness [15], demonstrating the broad impact of musical training on auditory skills.

Building on this evidence, several behavioural studies have examined how musical training sharpens auditory abilities. Karen and Merav [16] reported that musically trained children significantly outperformed their untrained peers in frequency discrimination, speech-in-noise perception, and word sequencing, suggesting that early and consistent exposure to music enhances auditory processing and cognitive-linguistic integration [16]. Similarly, Rammsayer and Altenmüller reported that musicians showed superior temporal acuity in rhythm perception, auditory fusion, and temporal discrimination tasks [13]. Interestingly, no group differences were observed in temporal generalisation, indicating that the benefits of music training are more pronounced for real-time perceptual timing than for memory-based temporal processing.

Further evidence highlights that musical training refines other key aspects of auditory processing. Johnson et al. demonstrated that musicians had better spectral profile analysis thresholds, with longer durations of training correlating with superior performance, pointing to enhanced sensitivity to spectral cues [7]. Likewise, Kumar et al. found that both vocal and instrumental musicians exhibited enhanced temporal resolution and discrimination abilities, with performance improving proportionally with years of practice [17]. Reinforcing the concept of auditory plasticity, Jain et al. revealed that even short-term musical training was sufficient to produce significant gains in speech perception in noise, underscoring how quickly the auditory system adapts to structured auditory experiences [14].

Neurophysiological evidence further supports these behavioural findings, as Zendel and Alain [18] demonstrated that musicians are more adept at segregating simultaneous sounds, particularly when subtle harmonic mistuning is present. This heightened ability was supported by stronger and earlier object-related negativity (ORN) responses and a larger P400 wave, reflecting enhanced perceptual and neural mechanisms for auditory scene analysis.

Collectively, these findings suggest that musical training induces neuroplastic changes within the auditory pathways and strengthens cognitive mechanisms for processing auditory information. These adaptations enhance the brain’s ability to analyse complex acoustic inputs, resulting in measurable improvements in temporal, spectral, and speech-in-noise perception.

In a similar context, dance training, with its deep integration of movement and music, has been shown to positively influence auditory processing. The synchronization of body movements with rhythmic cues requires complex spatial, temporal, and motor coordination [19]. Over time, such regular and intensive practice has been shown to strengthen neural pathways involved in cognitive functions, leading to improvements in auditory attention, working memory, and temporal resolution [7,20,21]. These effects have been widely studied and reported in dancers trained in Bharatanatyam, a classical danceform originating from Tamil Nadu in the 4th century [22], which combines intricate motor sequences with intensive auditory and rhythmic stimulation. Expanding on this relationship between dance and auditory processing, behavioural studies have consistently shown measurable enhancements in dancers’ auditory skills.

Silva et al. [20] demonstrated that dancers outperformed non-dancers on gap detection tasks, indicating that the integration of auditory and motor skills strengthens the neural mechanisms underlying temporal processing. Similarly, Pisharody et al. [23] found that professional dancers exhibited lower thresholds in both gap detection and temporal modulation transfer function tasks, reinforcing the idea that sustained rhythmic practice through dance sharpens temporal resolution and auditory sensitivity. Physiological evidence further supports these behavioural findings. Johnson et al. [7] reported that Bharatanatyam dancers exhibited greater contralateral suppression of otoacoustic emissions, indicating enhanced functioning of the efferent auditory pathway. This improvement may stem from the combined effects of rigorous motor training and musical instruction, which together promote greater plasticity of the auditory nerve system. Similarly, Prakash et al. [21] found that Bharatanatyam dancers demonstrated enhanced auditory working memory, with higher scores and faster response times in task performance. These findings suggest neuroplastic changes in the auditory and motor cortices, driven by the demands of intensive auditory–motor coordination inherent in dance training. Long-term Bharatanatyam training appears to foster neuroplastic adaptations within the auditory pathways and higher-order cognitive networks. This auditory–motor integration not only enhances temporal and spectral processing but also improves the brain’s capacity to analyse and respond to complex auditory inputs. Consequently, such adaptations provide dancers with a distinct advantage in advanced auditory skills, including auditory streaming, attentional control, and precise synchronisation with dynamic auditory cues.

While studies have explored enhanced auditory streaming abilities in formally trained musicians [7,18,24], there is a notable gap in the literature regarding the objective assessment of auditory stream segregation in formally trained Bharatanatyam dancers, highlighting the need for such investigations. Musicians have consistently demonstrated superior performance in tasks involving pitch discrimination, temporal processing, and speech-in-noise perception, which enhances their ability to separate complex auditory streams [7]. Similarly, Bharatanatyam dancers, though not primarily focused on musical notes or pitch variations, are continuously exposed to rhythmic patterns and musical beats. Their training demands precise synchronisation of movements and facial expressions with complex rhythmic cues, providing them with a unique form of dual auditory–motor exposure. This intense engagement with timing and rhythm may offer dancers comparable, if not distinct, advantages in auditory processing. Understanding these parallels and distinctions between musicians and dancers can provide deeper insights into how different forms of auditory training influence auditory stream segregation and speech-in-noise perception, reinforcing the need for comparative studies between these two trained populations.

In addition to these behavioural and perceptual aspects, auditory training and experience are known to modulate hemispheric specialisation and inter-hemispheric asymmetry in auditory processing. Evidence suggests that musicians exhibit stronger left-hemispheric dominance for temporal and speech-related cues and enhanced right-hemispheric activation for spectral and melodic information [9,25]. Such lateralised auditory processing patterns reflect experience-dependent neural plasticity that may differ across forms of auditory and motor training. Investigating laterality patterns through indices such as the laterality index provides insights into auditory skills. Comparative examination of laterality in musicians and dancers could, therefore, elucidate how different types of auditory–motor experiences shape cerebral asymmetry.

Despite the available evidence, limited studies have examined auditory stream segregation, speech-in-noise perception, and hemispheric asymmetry in Carnatic musicians and Bharatanatyam dancers compared to non-trained individuals. Thus, the present study aimed to address this gap by assessing and comparing the auditory stream segregation, speech-in-noise perception, and inter-hemispheric asymmetry (using the laterality index) in Carnatic musicians, Bharatanatyam dancers, and non-trained individuals. This study also sought to determine whether the duration of formal training influences these auditory performance measures, thereby providing a comprehensive understanding of how different forms of auditory exposure and training shape auditory processing and cerebral organization.

## 2. Materials and Methods

### 2.1. Participants

A total of 105 participants, aged between 18 and 30 years, were recruited for the study using purposive sampling. Participants were recruited from local music academies, Bharatanatyam dance institutes, and the general student population of Karnatak University. The study employed a prospective experimental design, wherein all participants were enrolled and assessed following predefined procedures. All the participants were right-handed individuals. The participants were divided into three groups. Group I comprised 35 formally trained Carnatic vocalists (mean age = 24.23 ± 1.93 years), each with a minimum of five years of academic training and at least 10 h of weekly practice. Group II included 35 Bharatanatyam dancers (mean age = 23.88 ± 2.85 years), each with a minimum of five years of formal dance training and active practice of at least 10 h per week. Group III consisted of 35 individuals (mean age = 23.08 ± 1.86 years) with no formal training in either music or dance. None of the participants in Group III reported habitual interests in music or dance performance, nor any specialized training in activities such as athletics, chess, or abacus during childhood, which could potentially have demonstrated enhanced auditory attention. Across all groups, none of the participants reported a history of otological symptoms (e.g., ear pain, tinnitus, vertigo), exposure to noise or ototoxic medication, nor any neurological disorders, habitual alcohol consumption, or smoking.

### 2.2. Instrumentation

A calibrated dual-channel diagnostic audiometer (GSI Audiostar Pro, Eden Prairie, MN, USA) with TDH 39 supra-aural headphones (Telephonics, Santa Ana, CA, USA) and a Radio Ear B-71 bone vibrator was used to obtain pure tone audiometry (PTA) thresholds and Speech Identification Scores (SIS). Tympanometry and acoustic reflex thresholds (ART) were measured using a calibrated GSI Tympstar middle ear analyzer to rule out middle ear pathology. Distortion product otoacoustic emissions (DPOAEs) were recorded using an Otodynamics ILOV6 Echoport system (Otodynamics, Hatfield, UK) to verify normal cochlear outer hair cell function.

For the auditory stream segregation task, MATLAB (R2014a) with a psychoacoustics toolbox utilizing the maximum likelihood procedure (MLP) was installed on a Lenovo 81D2 Ryzen 5 computer (Lenovo, Beijing, China). Stimuli were delivered through calibrated Sennheiser HD 569 headphones (Sennheiser, Wedemark, Germany). The Kannada version of the Quick Speech-in-Noise test (QuickSIN) was used to assess speech-in-noise performance. The stimuli were normalized and were presented at 60 dB SPL through a calibrated audiometer.

Speech-in-noise perception was assessed using the Kannada version of the Quick Speech-in-Noise test (QuickSIN). The QuickSIN test was selected because it provides a rapid and ecologically valid estimate of speech perception in multi-talker babble, a listening condition that closely resembles everyday communication environments. Unlike adaptive sentence-based tests such as the Hearing-in-Noise Test (HINT), which primarily assess speech reception thresholds under relatively steady noise conditions, QuickSIN employs fixed signal-to-noise ratios with increasing task difficulty, enabling efficient estimation of SNR-50 across a wide range of listener abilities. In addition, compared to word-based measures such as the Word-in-Noise (WIN) test, QuickSIN places greater demands on sentence-level processing and contextual integration, which are particularly relevant for examining training-related differences in central auditory processing. The speech stimuli were normalized and presented at 60 dB SPL through a calibrated audiometer.

### 2.3. Procedure

Participants first underwent a comprehensive preliminary evaluation that included a detailed case history focusing on otological/audiological symptoms and relevant life events, followed by otoscopic examination to rule out external ear pathology and confirm eligibility.

Pure tone thresholds were measured for octave and mid-octave frequencies for air conduction (250–8000 Hz) and bone conduction (250–4000 Hz) using the modified Hughson-Westlake procedure [26]. Individuals meeting the criterion of pure-tone averages ≤ 15 dB HL at all frequencies, with air-bone gaps not exceeding 10 dB, were included. Speech Identification Scores (SIS) were obtained using phonemically balanced (PB) words in Kannada [27]. All the participants had SIS ≥ 95% in quiet. Tympanometry and ipsilateral/contralateral acoustic reflexes were evaluated at 500, 1000, 2000, and 4000 Hz to confirm normal middle ear function. All participants demonstrated acoustic reflex thresholds within normal sensation levels.

DPOAEs were recorded at six points per octave between 1 kHz and 6 kHz to verify normal outer hair cell integrity. Participants met the signal-to-noise ratio (SNR) pass criterion of +6 dB for the majority of frequencies. All audiological assessments were performed in a sound-treated audiometric booth that complied with ANSI standards.

The Profile Analysis test within MATLAB software, installed with the psychoacoustics toolbox that implements the maximum likelihood procedure [28], was used to evaluate each participant’s auditory stream segregation sensitivity. Profile analysis thresholds were measured using customised MATLAB code at an intensity level of 60 dB SPL. The stimuli were delivered through calibrated Sennheiser HD 569 headphones connected to a Lenovo 81D2 Ryzen 5 computer in a sound-treated room. Participants were presented with three blocks of auditory stimuli, each containing tones with five harmonics. In the reference blocks, all five harmonics had identical amplitudes, whereas in the target block, the amplitude of the third harmonic was modulated, creating a distinct timbre. The test was administered for four frequencies (250, 500, 750, and 1000 Hz), with the order of frequency presentation randomised across participants. Each participant’s task was to identify the tone with the unique timbre in a three-alternative forced-choice paradigm.

The QuickSIN consisted of seven lists, with each list containing seven sentences. Each sentence included five target words. The lists were presented in the presence of an eight-talker competing speech babble at different SNR levels ranging from +8 dB to −10 dB in 3 dB steps. Based on the correct identification of the target words in the presence of competing babble, total scores were obtained for each discrete SNR level, and the SNR 50 was calculated. The SNR-50 value, defined as the signal-to-noise ratio corresponding to 50% speech recognition accuracy, was estimated using a customised MATLAB code implementing the Spearman-Kärber method [29] expressed as

$$SNR_{50}=L_{max}-d\times \left(\sum p_{i}-\frac{1}{2}\right)$$

where $L_{max}$ is the highest signal-to-noise ratio presented, *d* is the step size between successive SNR levels, and $p_{i}$ represents the proportion of correctly identified target words at each SNR level.

Practice trials were provided before testing to familiarise participants with the task, and these were not included in the final scoring. The stimuli were presented to right ear alone, left ear alone, and under binaural conditions to obtain the ear effects and laterality index. To control any possible order effects, the order of these presentations was randomised among the participants.

To assess cerebral asymmetry, the Laterality Index (LI) was calculated for each participant using the following equation:

$$LI=\frac{\left(R-L\right)}{\left(R+L\right)}$$

where *R* represents the SNR-50 value obtained for the right ear and *L* represents the SNR-50 value obtained for the left ear. Positive *LI* values indicate a right-ear advantage, whereas negative values indicate a left-ear advantage.

## 3. Results

The data were analysed to check for normal distribution using the Shapiro–Wilk test for normality. The results indicated that the data were not normally distributed (*p* < 0.05). Hence, all inferential statistical analyses were performed using non-parametric tests.

### 3.1. Comparison of Spectral Profile Analysis Thresholds Across and Within the Groups

**Figure 1:**
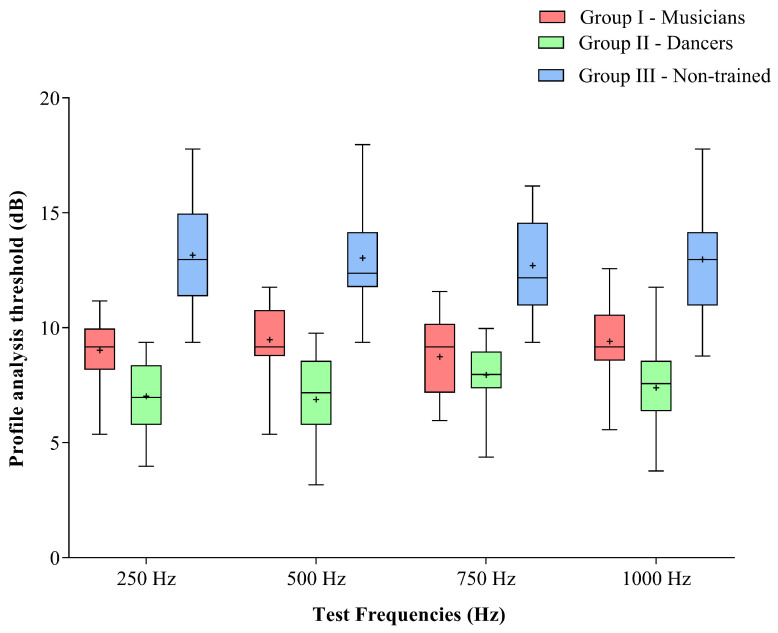
Box-and-whisker plot showing the median, 25th and 75th percentiles, and the minimum and maximum values of spectral profile analysis thresholds obtained at different test frequencies for musicians, dancers, and non-trained individuals. Note: ‘+’ indicates the mean value.

The dancers (Group II) demonstrated the lowest median thresholds, followed by the musicians (Group I) and the non-trained individuals (Group III). The median, quartiles, and minimum and maximum values of the spectral profile analysis thresholds obtained by the three groups for each test frequency are presented in Figure 1.

To examine the statistical significance of these observations, the thresholds obtained for each frequency were compared across the groups using the Kruskal–Wallis H test. The results revealed significant differences among the three groups at all test frequencies (*p* < 0.001).

Pairwise comparisons were then conducted using the Mann–Whitney U test with a Bonferroni-adjusted alpha value of 0.017 as the significance level. The results showed that the dancers exhibited significantly lower thresholds than the musicians at all test frequencies (*p* < 0.001), except at 750 Hz, where no significant difference was observed (*p* > 0.05). Comparisons of the dancers’ and musicians’ thresholds with those of the non-trained group revealed significantly lower thresholds for both trained groups at all four test frequencies (*p* < 0.001). The results of the pairwise comparisons, along with the calculated effect sizes, are presented in Table 1.

An additional exploratory analysis was conducted to examine whether the spectral profile analysis thresholds varied across the test frequencies within each group using the Friedman test. The results revealed no significant differences in thresholds across the four test frequencies for any of the three groups (*p* > 0.05).

### 3.2. Comparison of Speech-in-Noise Performance Across Groups

**Figure 2:**
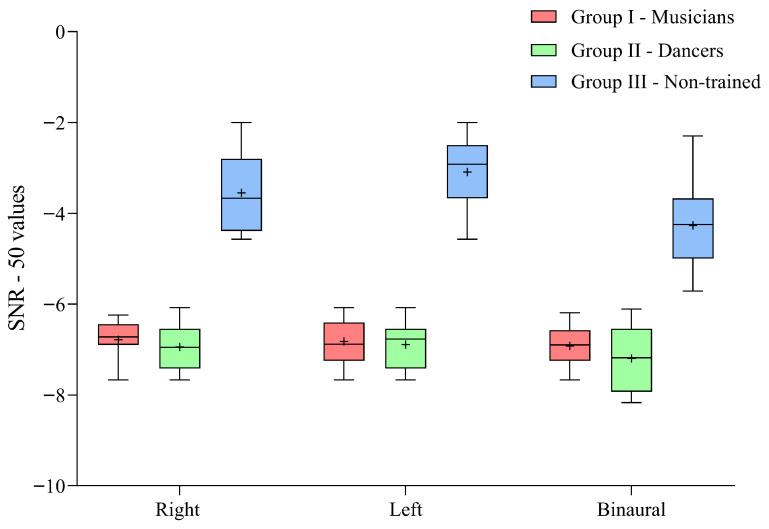
Box-and-whisker plot showing the median, 25th and 75th percentiles, and the minimum and maximum SNR-50 values obtained for the right ear, left ear, and binaural stimulus conditions in musicians, dancers, and non-trained individuals. Note: ‘+’ indicates the mean value.

The descriptive analysis revealed lower SNR-50 values indicating lesser deterioration of speech understanding performance in competing noise for musicians and dancers compared to non-trained individuals across the right, left, and binaural stimulus conditions. Dancers exhibited slightly lower SNR-50 values (better speech in noise performance) than musicians for the binaural and right-ear conditions, whereas musicians showed marginally higher values than dancers for the left-ear condition. The median binaural scores were lower compared to right and left ear scores for all three groups. The descriptive statistical results for the SNR-50 values obtained by the three groups under different stimulus conditions are presented in Figure 2.

The SNR-50 values for the right ear, left ear, and binaural stimuli were compared across the groups using the Kruskal–Wallis H test. The results revealed a significant difference between the groups (*p* < 0.001). To further examine the differences in speech-in-noise performance across groups, pairwise comparisons were conducted using the Mann–Whitney U test with a Bonferroni-adjusted alpha level of 0.017. The findings indicated significantly lower SNR-50 values for both musicians and dancers compared to the non-trained group across all three stimulus conditions (*p* < 0.001). However, no significant differences were observed between musicians and dancers for any of the three stimulus conditions (*p* > 0.05). The results of the pairwise comparisons, along with the corresponding effect sizes, are presented in Table 2.

### 3.3. Relationship Between Spectral Profile Analysis Thresholds and Speech-in-Noise Performance

To examine the relationship between spectral profile analysis thresholds and speech identification performance in noise, a Spearman’s rank-order correlation was conducted. Since the spectral profile analysis thresholds were obtained using binaural stimuli presentation, only the binaural SNR-50 values were opted to run the correlation. The results revealed no significant correlation between SNR-50 values and profile analysis thresholds for the musician and dancer groups (*p* > 0.05). In contrast, for the non-trained individuals, a significant correlation was observed at 250 Hz and 1000 Hz (*p* < 0.05), whereas the correlations at 500 Hz and 750 Hz were not significant. A positive correlation was observed between these parameters, indicating that lower spectral profile analysis thresholds were associated with lower SNR-50 values (reflecting better speech-in-noise performance). The findings of the Spearman correlation analysis for the three groups are presented in Table 3.

### 3.4. Comparison of Cerebral Asymmetry in Speech-in-Noise Performance Across the Three Groups

To compare cerebral asymmetries, the Laterality Index (LI) was calculated for each participant and analysed across the groups. The *LI* was computed using the formula $LI=\frac{R-L}{R+L}$, where *R* represents the SNR-50 value for the right ear and *L* represents the SNR-50 value for the left ear. A positive *LI* value indicates a right-ear advantage, whereas a negative *LI* value reflects a left-ear advantage. Since higher SNR-50 values correspond to poorer speech-in-noise performance, the interpretation of the *LI* considered this inverse relationship.

**Figure 3:**
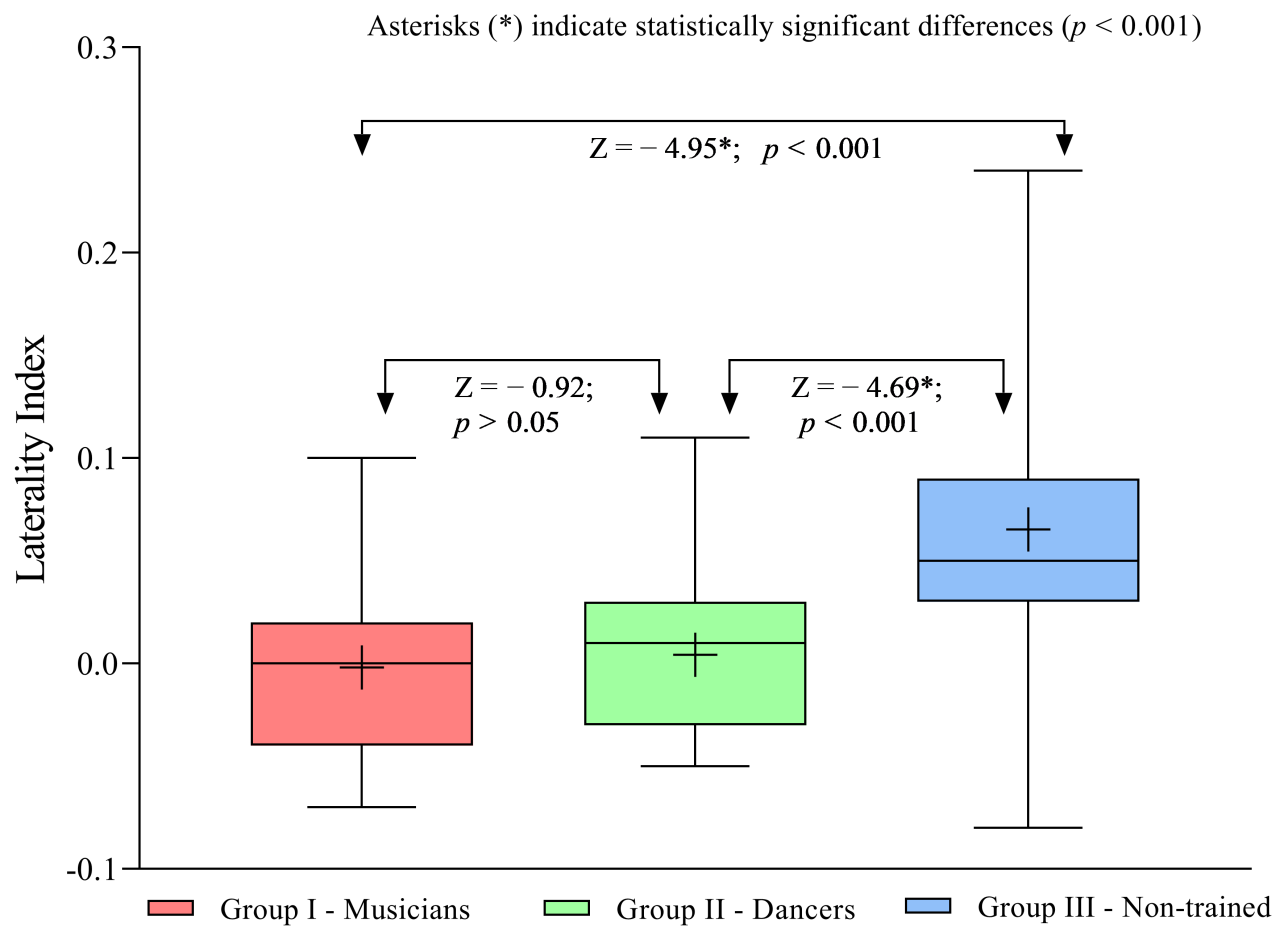
Box-and-whisker plot showing the median, 25th and 75th percentiles, and the minimum and maximum values of the Laterality Index (LI) obtained for musicians, dancers, and non-trained individuals. Positive LI values indicate a right-ear advantage, whereas negative values indicate a left-ear advantage. Note: ‘+’ indicates the mean value.

The *LI* values obtained for the three groups were compared using the Kruskal–Wallis H test, which revealed a significant difference in laterality indices across groups (*p* < 0.001). To identify specific group-wise differences, pairwise comparisons were conducted using a Bonferroni-adjusted alpha value of 0.017. No significant difference was observed between the laterality indices of the musician and dancer groups; however, both groups significantly differed from the non-trained group. An interesting observation was that the musician and dancer groups exhibited markedly reduced or negligible laterality compared to the non-trained group, indicating minimal cerebral asymmetry in these populations (*p* < 0.001). The laterality indices for the three groups and the results of pairwise comparisons are presented in Figure 3.

### 3.5. Association Between Extent of Training and Auditory Performance Measures

The median and quartile values were obtained for the duration in training in the years and average weekly practice hours reported by the musicians and dancers. the findings are provided in the Table 4.

To assess the association between the extent of training and different auditory performance measures, a Spearman correlation was carried out. The duration of years of practice and the average number of hours of practice reported by the participants during the interview were taken as the predictor variables, while the spectral profile analysis thresholds, binaural SNR 50 values, and laterality index were considered as the outcome measures. These analyses were conducted independently for the musician and dancer groups.

For the musicians, significant negative correlations were observed between the duration of training and the spectral profile analysis thresholds at 250 Hz (*p* < 0.01) and 750 Hz (*p* < 0.05), indicating that longer training duration was associated with better (lower) threshold performance at these frequencies. No significant correlations were observed at other test frequencies, SNR 50, or laterality index. The weekly practice hours did not show any significant correlations with any of the auditory outcome measures.

For the dancers, significant negative correlations were found between the duration of training and the spectral profile analysis thresholds at 250 Hz (*p* < 0.01) and 500 Hz (*p* < 0.05), as well as the binaural SNR 50 (*p* < 0.01). These findings suggest that individuals with a longer period of dance training demonstrated better spectral resolution and speech perception in noise abilities. No other significant associations were observed for other test frequencies, laterality index, or weekly practice hours.

The results of the correlation analysis obtained for the musician and dancer groups are summarised in Table 5.

## 4. Discussion

The present study aimed to evaluate and compare spectral profile analysis thresholds (SPAT) and interhemispheric asymmetry in processing speech in noise among trained musicians, trained dancers, and non-trained controls. The findings revealed enhanced auditory abilities reflected by lower SPAT and SNR-50 values in musicians and dancers compared to age-matched non-trained individuals. To varying extents, both musicians and dancers undergo rigorous and sustained training as part of their long-term practice. Such prolonged training is known to induce neuroplastic changes in auditory and associated cortical regions over time. Therefore, the auditory advantages observed in these two groups could be attributed to training-induced neuroplasticity.

### 4.1. Spectral Profile Analysis and Speech-in-Noise Perception in Musicians and Dancers

Supporting this interpretation, extensive literature has shown that professional musicians exhibit enhanced auditory skills across several domains, including auditory memory [11], temporal processing [30], pitch discrimination [17], and auditory attention [24]. Musicians also display superior spectro-temporal acuity in identifying and discrimination of speech stimulus [24,31] and demonstrate better extraction of speech cues in challenging listening environments [32]. These advantages have been attributed to narrower auditory filters and sharper tuning curves that allow more efficient perception of spectral timbre and speech cues even under degraded listening conditions [33,34]. Electrophysiological evidence further supports these findings, with studies reporting earlier latencies and enhanced brainstem responses in musicians, reflecting more efficient early sensory encoding of sound [35].

Similarly, dance training has been shown to produce comparable auditory and cognitive benefits. Prakash et al. [21] reported enhanced auditory working memory and shorter reaction times in trained Bharatanatyam dancers, while Johnson et al. [7] documented greater contralateral suppression of otoacoustic emissions, indicating stronger medial olivocochlear functioning associated with long-term dance practice. Dance training induces significant neuroplastic changes through its integration of physical, cognitive, auditory, and motor components [36]. Neuroimaging studies indicate increased brain volumes in regions supporting motor control, sensory integration, and memory in dancers—surpassing effects observed in traditional fitness activities [37]. Additional findings of lower gap detection thresholds in trained dancers [38] and improved multisensory integration and attentional control [39] further support enhanced temporal and auditory processing in this population.

Collectively, these findings provide converging evidence for training-induced auditory neuroplasticity and suggest that long-term musical or dance training enhances auditory processing abilities and attentional mechanisms.

Upon further pairwise comparison, dancers outperformed musicians by demonstrating significantly lower spectral profile analysis thresholds. This advantage may be attributed to the dual mode (sensory and motor) demands of dance practice, which integrates auditory and motor training over sustained periods. However, no significant difference emerged in the speech-in-noise measures between the two groups, possibly due to a floor effect, wherein both groups demonstrated very low (i.e., better) SNR-50 scores, limiting the sensitivity to detect subtle between-group differences. In this context, speech-in-noise tasks involving noise-in-noise masking, fluctuating maskers, or spatially separated competing talkers may provide greater sensitivity to subtle performance differences among expert listeners. Such paradigms impose higher perceptual and cognitive demands and have been shown to better differentiate individuals with advanced auditory skills. Therefore, the limited variability observed in SNR-50 scores in the present study likely reflects ceiling-level performance relative to task difficulty rather than an absence of training-related effects.

In summary, both the musician and dancer groups demonstrated clear auditory advantages over non-trained individuals, with Bharatanatyam dancers exhibiting some additional benefits relative to Carnatic musicians [30].

### 4.2. Relationship Between Spectral Profile Analysis Thresholds and Speech Perception in Noise Abilities

When assessing the correlation between spectral profile analysis thresholds and SNR-50 values (both obtained under binaural listening conditions), no significant correlations were observed for the musician or dancer groups overall. However, significant positive correlations emerged at two test frequencies (250 Hz and 1000 Hz), indicating that individuals with lower profile analysis thresholds demonstrated better speech perception in noise. This finding aligns with the notion that enhanced spectral coding contributes to more effective speech understanding in competing noise. As the remaining test frequencies did not show significant associations, these observations should be interpreted cautiously and validated with a larger sample. A possible explanation for the limited correlations is the previously mentioned floor effect in SNR-50 scores among the musician and dancer groups, which may have reduced the variability required to detect meaningful relationships between the two measures.

### 4.3. Comparison of Cerebral Asymmetry in Musicians, Dancers and Non-Trained Individuals

The cerebral asymmetry in processing speech stimuli in the presence of noise was calculated using the laterality index (LI), where positive LI values indicate left hemisphere dominance or a right-ear advantage, and negative LI values indicate right hemisphere dominance or a left-ear advantage. As described in the Methods section, all participants were right-handed individuals, implying that they were likely to have left-hemisphere dominance. Upon comparing the right versus left-ear speech-in-noise scores (as reflected by the SNR-50 values), a positive LI was observed, indicating significantly better performance when the stimuli were presented to the right ear. Behavioral studies using dichotic and monaural speech tasks have consistently reported a right-ear advantage (REA) for verbal stimuli in normal young adults, reflecting the functional advantage of the left hemisphere for speech processing [40]. Evidence specific to speech-in-noise perception also indicates an REA: several investigations have found better right-ear performance on speech-in-noise tests, supporting the behavioral finding of right-ear superiority under acoustically challenging conditions [41]. Recent work further shows that the REA persists in more naturalistic and dynamic “cocktail-party” paradigms, reinforcing the idea that right-ear/right-hemisphere asymmetries are observable beyond classic dichotic tasks [42]. Complementing these behavioral data, a large body of functional neuroimaging demonstrates predominant left-hemisphere specialization for language in typical, right-handed adults, showing a pattern that stabilizes in late adolescence and early adulthood and providing the neural substrate for the commonly observed right-ear behavioral advantage [43]. Together, these behavioural and imaging findings offer converging support for the positive LI (right-ear advantage/left-hemisphere dominance) observed in our right-handed sample and for the significantly higher right-ear SNR-50 performance reported above.

Contrastingly, and interestingly, this asymmetry (as indicated by the laterality indices) was minimal or entirely absent in the musician and dancer groups. A possible explanation for the reduced hemispheric dominance in these populations is the neuroplastic changes occurring within the corpus callosum and auditory cortical regions of both hemispheres. Supporting this interpretation, several studies have reported that individuals with long-term musical or dance training exhibit enhanced interhemispheric connectivity and functional reorganization in auditory and related neural networks.

Several empirical studies support the notion that long-term sensorimotor training can alter typical hemispheric asymmetries. Behavioral and interhemispheric-transfer studies have reported reduced lateralization or more symmetric interhemispheric transfer in musicians compared to non-musicians, consistent with enhanced callosal connectivity and more bilateral auditory processing [44,45]. Electrophysiological and EEG connectivity studies further show that musicians often recruit more bilateral networks (reduced asymmetry) during complex auditory tasks, including language-related and music-related processing [46]. Similarly, imaging and white-matter studies indicate neuroanatomical adaptations in dancers and musicians, including changes in corpus callosum morphology and altered white-matter diffusivity in sensorimotor/auditory pathways that plausibly support reduced functional asymmetry in these trained populations [45]. Some music/dance cohorts have also shown tendencies toward right-hemisphere predominance for particular visuomotor or rhythmic tasks, suggesting that task demands and training modality can produce diverse lateralization outcomes across performer populations [37]. Together, these behavioral, electrophysiological, and imaging findings provide converging evidence that long-term musical or dance training can diminish typical left-hemisphere dominance for auditory/verbal tasks, which can explain the minimal or absent LI observed in our musician and dancer groups.

### 4.4. Relationship Between Training Duration and Auditory Performance in Musicians and Dancers

The SPAT values obtained at 250 Hz and 750 Hz showed a negative correlation with the number of years of musical training, while dancers demonstrated a similar negative association at 250 Hz and 500 Hz. Additionally, dancers also exhibited a negative correlation between their binaural SNR-50 values and years of training. Apart from these findings, no other significant correlations were observed between any of the auditory variables and training duration. Likewise, weekly practice hours did not show any systematic relationship with the auditory measures assessed in this study.

Although no strong correlations emerged between training hours or years and the evaluated parameters, the hypothesis that longer training durations promote greater neuroplastic changes within the auditory and associated pathways remain theoretically sound. It must be acknowledged, however, that the current methodology may lack the sensitivity to detect such associations primarily due to the previously discussed floor effects observed in the trained groups’ scores. To better validate this assumption, future studies should consider adopting a cross-sectional design that includes participants ranging from beginners to advanced experts (spanning less than one year to more than ten years of training and consistent practice).

### 4.5. Comparison with Prior Literature and Contribution of the Present Study

Previous investigations have consistently demonstrated enhanced auditory stream segregation and spectral processing abilities in musicians compared to non-musicians, particularly using profile analysis and related psychoacoustic paradigms [7,18,24]. The present findings align with these reports by confirming significantly lower spectral profile analysis thresholds in Carnatic musicians relative to non-trained individuals. However, extending earlier work, the current study demonstrates that Bharatanatyam dancers exhibit equal or superior spectral discrimination abilities compared to musicians, highlighting that intensive rhythmic motor training may confer auditory benefits comparable to, or exceeding, those observed with formal musical training.

While earlier studies have primarily focused on musicians when examining speech-in-noise perception advantages [11,12,13,14,32], fewer investigations have explored such abilities in dancers. Our results corroborate prior findings of enhanced speech-in-noise perception in musicians but further extend this literature by demonstrating comparable improvements in Bharatanatyam dancers across monaural and binaural listening conditions. This suggests that sustained auditory–motor synchronization inherent in dance training may enhance speech perception in noisy environments, even in the absence of explicit pitch based musical training.

Regarding hemispheric asymmetry, previous research using dichotic listening and neuroimaging paradigms has reported altered or reduced lateralization in musicians, attributed to enhanced interhemispheric connectivity and bilateral auditory network recruitment [44,45,46]. The present study extends these findings by demonstrating reduced laterality indices during a functional speech-in-noise task in both musicians and dancers. Importantly, this observation expands existing evidence by showing that reduced hemispheric asymmetry is not limited to musicians but may also emerge from long-term dance training, suggesting a shared mechanism of experience-dependent auditory–motor neuroplasticity.

Furthermore, although earlier studies have reported associations between years of training and auditory performance [7,17], the present findings indicate that such relationships may be frequency- and task-specific and susceptible to floor effects in highly trained cohorts. By examining both training duration and weekly practice hours across two distinct trained populations, this study provides a more nuanced understanding of how different dimensions of training experience relate to auditory outcomes.

The present study advances the existing literature by offering the first comprehensive comparison of auditory stream segregation, speech-in-noise perception, and cerebral asymmetry among Carnatic musicians, Bharatanatyam dancers, and non-trained individuals. These findings underscore that structured auditory–motor experiences, whether music- or dance-based, can lead to overlapping yet distinct patterns of auditory enhancement and neural organization.

### 4.6. Limitations

- The cross-sectional design limits causal inference regarding the effects of music or dance training on auditory processing, precluding conclusions about training- induced plasticity.
- The trained groups primarily consisted of highly experienced performers, resulting in restricted performance variability and probable floor effects in some of the auditory measures.
- The sample was confined to young, right-handed adults, limiting the generalisability of the findings.
- Only Carnatic vocalists and Bharatanatyam dancers were examined, and therefore, the results may not extend to other musical traditions or dance forms.
- Influence of passive music exposure on auditory processing in the non-trained control group was not quantified.

### 4.7. Future Directions

- Longitudinal studies tracking individuals across different stages of musical or dance training are needed to establish causal links between training and auditory plasticity.
- Future work should include participants spanning a broader range of expertise, from novices to experts.
- Extending investigations to middle-aged and older adults would help evaluate the translational relevance of music and dance training for age-related auditory and cognitive decline.
- Incorporating neurophysiological and neuroimaging measures such as EEG, auditory brainstem responses, or functional MRI would help elucidate the neural substrates of reduced hemispheric asymmetry and enhanced auditory performance.
- Influence of passive music exposure on auditory processing in the non-trained control group was not quantified.

## 5. Conclusions

The present study demonstrated that long-term musical and dance training is associated with enhanced auditory processing, reflected in superior spectral profile analysis thresholds, better speech-in-noise perception, and reduced cerebral asymmetry compared to non-trained individuals. While both groups of trained performers showed clear auditory advantages, dancers exhibited marginally stronger spectral resolution abilities than musicians. The absence of strong correlations between training duration and auditory outcomes–likely influenced by floor effects in performance scores–suggests the need for more sensitive designs involving a wider range of training expertise. Quantitatively, both musicians and dancers demonstrated substantially lower spectral profile analysis thresholds and better speech-in-noise performance compared to non-trained individuals, with large effect sizes observed across frequencies and listening conditions. In addition, non-trained listeners showed a clear right-ear advantage during speech-in-noise perception, whereas musicians and dancers exhibited minimal laterality, reflecting reduced hemispheric asymmetry. These numerical trends consistently support the presence of training-related enhancements in auditory stream segregation, speech perception in noise, and functional auditory organization. Overall, the findings highlight the role of sustained sensorimotor–auditory training in shaping neural and behavioural auditory abilities and underscore the importance of considering training-related neuroplasticity when evaluating auditory performance across specialized populations. 

## Figures and Tables

**Table 1:** Results of Mann–Whitney U pairwise comparisons of spectral profile analysis thresholds (SPAT) across test frequencies (250, 500, 750, and 1000 Hz) among musicians, dancers, and non-trained controls. The table highlights the magnitude and consistency of training-related group differences, with effect sizes indicating stronger spectral stream segregation abilities in trained groups, particularly dancers, compared to non-trained listeners. * significant at *p* < 0.05.

Groupwise Comparison	Test Statistic Values			
	250 Hz	500 Hz	750 Hz	1000 Hz
**Musicians vs. Dancers**				
*U*	193.00	200.50	439.50	227.00
*Z*	−4.84 *	−4.74 *	−1.87	−4.42 *
*r*	0.58	0.57	0.22	0.53
**Musicians vs. Controls**				
*U*	57.50	72.00	66.00	103.00
*Z*	−6.53 *	−6.36 *	−6.42 *	−5.99 *
*r*	0.78	0.76	0.77	0.72
**Dancers vs. Controls**				
*U*	6.00	5.00	6.00	17.50
*Z*	−7.19 *	−7.20 *	−7.18 *	−7.05 *
*r*	0.86	0.86	0.85	0.84

**Table 2:** Results of pairwise group comparisons for SNR-50 thresholds across right, left, and binaural listening conditions using the Mann–Whitney U test. Effect sizes (*r*) were computed using the formula $r=Z/\sqrt{N}$. Asterisks (*) indicate a significance level of p<0.001.

Comparison	Right	Left	Binaural
**Musicians vs. Dancers**			
*U*	469.50	461.00	536.00
*Z*	−1.51	−1.61	−0.71
*r*	0.18	0.19	0.08
**Musicians vs. Controls**			
*U*	0.00	0.00	0.00
*Z*	−7.15 *	−7.11 *	−7.20 *
*r*	0.85	0.85	0.86
**Dancers vs. Controls**			
*U*	0.00	0.00	0.00
*Z*	−7.21 *	−7.24 *	−7.26 *
*r*	0.86	0.86	0.86

**Table 3:** Spearman’s rank-order correlation coefficients (ρ) between spectral profile analysis thresholds and binaural SNR-50 values across test frequencies for musicians, dancers, and non-trained controls. Significant associations were observed primarily in the non-trained group, suggesting greater dependence on basic spectral cues for speech-in-noise perception in untrained listeners. * significant at *p* < 0.05.

Group	Statistic	Test Frequencies			
		250 Hz	500 Hz	750 Hz	1000 Hz
Musicians	Spearman’s ρ	0.33	−0.19	0.05	−0.02
	*p*	0.06	0.28	0.79	0.92
Dancers	Spearman’s ρ	−0.03	0.02	−0.15	−0.26
	*p*	0.86	0.89	0.39	0.14
Non-trained	Spearman’s ρ	0.47 *	−0.02	0.33 *	0.31
	*p*	<0.01	0.91	0.04	0.07

**Table 4:** Median and quartile values for duration of training (years) and weekly practice (hours) for musician and dancer groups.

Variable	Group	Quartiles		
		Q1	Median	Q3
Training duration (years)	Musicians	6	7	8.25
	Dancers	7	9	10
Weekly practice (hours)	Musicians	9	11	13.25
	Dancers	4	5	15

**Table 5:** Spearman’s correlation coefficients (ρ) between training experience and auditory performance measures for musician and dancer groups. Asterisks (*) and (**) indicate significance levels of p<0.05 and p<0.01, respectively.

Group	Predictor	Profile Analysis (Hz)				SNR-50	Laterality
		250	500	750	1000		
Musicians	Training years						
	ρ	−0.52 **	−0.04	−0.39 *	−0.30	−0.04	−0.05
	*p*	0.00	0.84	0.04	0.09	0.83	0.78
	Practice hours						
	ρ	−0.28	0.08	−0.16	−0.21	−0.24	−0.29
	*p*	0.11	0.64	0.38	0.23	0.17	0.09
Dancers	Training years						
	ρ	−0.51 **	−0.39 *	−0.24	−0.14	−0.53 **	−0.01
	*p*	0.00	0.02	0.16	0.43	0.00	0.97
	Practice hours						
	ρ	−0.09	0.07	−0.03	−0.03	−0.29	−0.20
	*p*	0.61	0.68	0.88	0.88	0.10	0.26

## Data Availability

The datasets generated during and/or analysed during the current study are not publicly available due to institutional research policies regarding data sharing but are available from the corresponding author upon reasonable request and subject to institutional approval.
